# Combining Acceleration Techniques for Low-Dose X-Ray Cone Beam Computed Tomography Image Reconstruction

**DOI:** 10.1155/2017/6753831

**Published:** 2017-06-05

**Authors:** Hsuan-Ming Huang, Ing-Tsung Hsiao

**Affiliations:** ^1^Medical Physics Research Center, Institute for Radiological Research, Chang Gung University and Chang Gung Memorial Hospital, Taoyuan 33302, Taiwan; ^2^Department of Nuclear Medicine and Neuroscience Research Center, Chang Gung Memorial Hospital, Taoyuan 33302, Taiwan; ^3^Department of Medical Imaging and Radiological Sciences and Healthy Aging Research Center, College of Medicine, Chang Gung University, Taoyuan 33302, Taiwan

## Abstract

**Background and Objective:**

Over the past decade, image quality in low-dose computed tomography has been greatly improved by various compressive sensing- (CS-) based reconstruction methods. However, these methods have some disadvantages including high computational cost and slow convergence rate. Many different speed-up techniques for CS-based reconstruction algorithms have been developed. The purpose of this paper is to propose a fast reconstruction framework that combines a CS-based reconstruction algorithm with several speed-up techniques.

**Methods:**

First, total difference minimization (TDM) was implemented using the soft-threshold filtering (STF). Second, we combined TDM-STF with the ordered subsets transmission (OSTR) algorithm for accelerating the convergence. To further speed up the convergence of the proposed method, we applied the power factor and the fast iterative shrinkage thresholding algorithm to OSTR and TDM-STF, respectively.

**Results:**

Results obtained from simulation and phantom studies showed that many speed-up techniques could be combined to greatly improve the convergence speed of a CS-based reconstruction algorithm. More importantly, the increased computation time (≤10%) was minor as compared to the acceleration provided by the proposed method.

**Conclusions:**

In this paper, we have presented a CS-based reconstruction framework that combines several acceleration techniques. Both simulation and phantom studies provide evidence that the proposed method has the potential to satisfy the requirement of fast image reconstruction in practical CT.

## 1. Introduction

Based on the theory of compressive sensing (CS) [1, 2], near optimal computed tomography (CT) image can be reconstructed from very few projection data. This new methodology indicates a potential for substantially reducing radiation dose. Over the past decade, many CS-based image reconstruction methods have been shown to improve image quality in low-dose CT [3–8]. Such methods are often referred to as total variation (TV). To solve the TV problem in the field of CT reconstruction, a two-step alternating minimization framework is commonly used. In the first step, a general iterative reconstruction algorithm is performed to reduce data discrepancy. In the second step, the TV of the reconstructed image is minimized by a gradient descent method. Despite a great improvement of image quality with CS-based reconstruction methods, both high computational load and slow convergence rate limit their practical use.

Recently, the one-step minimization scheme such as first-order gradient-projection backtracking-line search method [9] and gradient-projection Barzilai-Borwein method was found to converge faster than two-step alternating minimization scheme [9–11]. However, there are various ways to improve the convergence rate of the two-step alternating minimization scheme. For example, image reconstruction based on ordered subsets (OS) of projection data is a common acceleration technique used in emission tomography [12] and CT [13–15]. Using OS acceleration [12], data discrepancy can be reduced rapidly compared with non-OS methods. In addition to OS-type simultaneous algebraic reconstruction technique (SART) [15], other faster methods such as ordered subsets transmission (OSTR) algorithm [13] and ordered subsets convex (OSC) algorithm [14] were derived previously. It was also reported that OS-type reconstruction methods can be further accelerated using a bigger step size or a power factor [16–18]. Previous studies showed that accelerated OS-type algorithms using a power factor can converge three or even four times faster than conventional OS-type algorithms [16–18]. These methods, although originally used in emission tomography, can be directly applied for the CT reconstruction. Besides reducing data discrepancy, many different minimization techniques, including fast iterative shrinkage thresholding algorithm (FISTA) [19], TV minimization with dual dictionaries [20], anisotropic TV minimization [21], total difference (TD) with soft-threshold filtering (STF) [6, 8], weighted TD (WTD) minimization with STF [22], and TV minimization with half-threshold filtering [23], can be introduced to improve the convergence of the two-step alternating minimization scheme.

Although many acceleration techniques have been developed previously, a combination of acceleration techniques has not been completely studied. To investigate this, we present a reconstruction framework that applies some above-mentioned acceleration techniques to the two-step alternating minimization. Specifically, we implemented the TD minimization with STF (TDM-STF), which is one type of TV [6, 8, 24]. In the TDM-STF, the OSTR algorithm was chosen to accelerate the convergence. To further speed up the convergence of the proposed method, we applied the power factor [16–18] and the FISTA algorithm [19] to OSTR and TDM-STF, respectively. This study is different from our recent work [25] that investigated the feasibility of using the power factor [16–18] to accelerate the TV-based reconstruction [5]. The purpose of this paper is to study whether combining these techniques can further accelerate the convergence of the two-step alternating minimization. We used simulation and phantom data to evaluate the performance of the proposed algorithm.

## 2. Materials and Methods

### 2.1. CT Image Reconstruction Problem

According to the idea of CS [1, 2] and the TDM algorithm proposed by Yu and Wang [6], the CT image reconstruction problem is to solve the constrained convex optimization problem of the following form:

(1) minμ fμ=Hμ−b−22+ωTDμs.t. μ≥0,

where *μ* is the image estimate, *H* is the system matrix, $\overset{-}{b}$ is the measured projection data, *ω* is a regularization factor, and TD(*μ*) denotes the total difference of the image estimate defined as [6]

(2) TDμ=∑x,y,zdx,y,z=∑x,y,zμx,y,z−μx+1,y,z+μx,y,z−μx,y+1,z+μx,y,z−μx,y,z+1.

*d*_*x*,*y*,*z*_ in (2) is called a discrete difference transform (DDT) [6, 8]. Similar to the TV problem [3–5], the TD problem in (1) can be solved iteratively using a two-step alternating minimization scheme [6, 8]. The TDM-STF algorithm [6] was a two-step alternating minimization algorithm. In this study, we used the TDM-STF algorithm [6] to minimize (1). Our aim is to improve the convergence rate of the TDM-STF algorithm [6]. The TDM-STF algorithm and its accelerated algorithm are summarized in the following three sections, respectively. Finally, the implementation of the proposed reconstruction algorithm is summarized in a pseudocode.

### 2.2. TDM-STF and TDM-STF-FISTA

The STF algorithm proposed by Daubechies et al. [26] was originally developed to solve the linear inverse problems regularized by a sparsity constraint. Yu and Wang [6] adapted the STF method for CT image reconstruction and developed the TDM-STF algorithm to minimize (1). In brief, the TDM-STF method involves three steps. In the first step, the data-fidelity term (i.e., ‖*Hμ* − *g*‖_2_^2^) was minimized via a typical iterative reconstruction algorithm. In the second step, a soft-threshold filtration was performed on the DDT of the current image estimate (i.e., ${\hat{\mu }}_{j}^{k,l+1}$). In the third step, the inverse of DDT was computed to obtain image estimate. However, DDT is not invertible [6]. Instead, the second and third steps of the TDM-STF algorithm can be performed based on a pseudoinverse of DDT [6]:

(3) μ~x,y,zk,l+1=16qω,μ^x,y,zk,l+1,μ^x+1,y,zk,l+1+qω,μ^x,y,zk,l+1,μ^x,y+1,zk,l+1+qω,μ^x,y,zk,l+1,μ^x,y,z+1k,l+1+qω,μ^x,y,zk,l+1,μ^x−1,y,zk,l+1+qω,μ^x,y,zk,l+1,μ^x,y−1,zk,l+1+qω,μ^x,y,zk,l+1,μ^x,y,z−1k,l+1,

where *x*, *y*, and *z* denote the three-dimensional location of the voxel *j* and

(4) $$\begin{array}{c} q\left(\;\omega ,a,b\;\right)=\left\{\begin{array}{cc} \frac{a+b}{2}, & if\;\;\left|a-b\right|<\omega \\ a-\frac{\omega }{2}, & if\;\;\left(a-b\right)\geq \omega \\ a+\frac{\omega }{2}, & if\;\;\left(a-b\right)\leq -\omega , \end{array}\right. \end{array}$$

where *ω* is a threshold value. As pointed out by Liu et al. [8], the TDM-STF method can be further accelerated by using a portion of FISTA algorithm [19], which is performed by the following steps:

(5) μjnew=μ~x,y,zk,l+1,

(6) tk=0.5×1+1+4tk−12,

(7) μ−−jk,l+1=μjnew+tk−1−1×μjnew−μjoldtk,

(8) μjold=μjnew.

This accelerated technique is also called Nesterov's momentum algorithms [27, 28]. Note that *t*_0_ = 1 and *μ*_*j*_^old^ = 0 when *k* = 0.

### 2.3. Acceleration of OSTR Using a Power Factor *h*

In the first step of the TDM-STF algorithm, the data-fidelity term was minimized via a typical iterative reconstruction algorithm. To speed up the data-fidelity minimization, we used the OSTR algorithm [13]. The OSTR algorithm is chosen because it is a fast, efficient, and easily implemented algorithm [13]. Moreover, the OSTR algorithm [13] can be accelerated by a power factor *h* [16–18]. The accelerated OSTR (AOSTR) algorithm can be expressed as follows:

(9) μjk,l+1=μjk,l1+Lμjk,l∑iHijrib−i ∑i∈SlHijbie−∑jHijμjk,l−b−ih,

where *μ*_*j*_^*k*,*l*^ is the estimated attenuation coefficient at voxel *j* and at the *k*th iteration and *l*th subiteration, ${\overset{-}{b}}_{i}$ is the measured projection data at detector bin *i*, *b*_*i*_ is the blank scan at detector bin *i*, *r*_*i*_ = ∑_*j*_*H*_*ij*_, and *L* is the number of subsets. Note that the AOSTR algorithm becomes the OSTR algorithm when power factor *h* = 1. Using the Taylor series expansion, (9) can be approximated as follows:

(10) μjk,l+1=μjk,l+hL∑iHijrib−i ∑i∈SlHijbie−∑jHijμjk,l−b−i.

More details can be found in [16–18]. Interestingly, the AOSTR algorithm is the same as the OSTR algorithm with a fixed step size. However, in order to preserve the total counts of the forward projections [16–18], the reconstructed image updated by (10) is rescaled by the following equation:

(11) $$\begin{array}{c} {\hat{\mu }}_{j}^{k,l+1}=\mu_{j}^{k,l+1}\frac{\sum_{i\in S_{l}}\log\left(b_{i}/{\overset{-}{b}}_{i}\right)}{\sum_{i\in S_{l}}\sum_{j}H_{ij}\mu_{j}^{k,l+1}}. \end{array}$$

Note that the solution for (1) from the OSTR algorithm and its accelerated version is not exact, but approximate [29]. An initial condition of uniform image (*μ*_*j*_^0,0^) with a value of 0.0002 was used for all reconstructions.

### 2.4. AOSTR-TDM-STF-FISTA

In summary, we applied the AOSTR reconstruction rather than the conventional iterative reconstruction method in the first step of TDM-STF with FISTA, and the proposed method was called AOSTR-TDM-STF-FISTA. In fact, other combinations of OSTR, AOSTR, and FISTA into the TDM-STF method are possible. For example, the accelerations of OSTR-TDM-STF using a power factor on OSTR and FISTA on TDM-STF are called AOSTR-TDM-STF and OSTR-TDM-STF-FISTA, respectively. In addition to the proposed AOSTR-TDM-STF-FISTA, difference combinations of the acceleration methods are also explored in this study.

### 2.5. Implementation of AOSTR-TDM-STF-FISTA

Note that the implementation of AOSTR-TDM-STF-FISTA requires considerably more computation per iteration as compared with OSTR. This is due to the computation of the rescaling factor (i.e., (11)) and the TDM step (i.e., (3)) at each subiteration. To reduce the computation time, the TDM step was applied after the last subiteration of each iteration, indicating that STF is performed only once at each iteration of the AOSTR algorithm. Because of this implementation, the rescaling step can be combined with the forward projection of the next subiteration except for the last subiteration [16–18]. This means that the rescaling step is computed only at the end of each iteration (i.e., the last subiteration). Similarly, the FISTA algorithm is performed once per iteration rather than once per subiteration. Such modifications make the proposed method an efficient approach for CT reconstruction. The final practical and efficient implementation of AOSTR-TDM-STF-FISTA can be summarized in Pseudocode 1.

As reported by Kim et al. [28], OS-type reconstruction algorithms combined with FISTA become unstable when a large number of subsets were used. To prevent an unstable convergence behavior, FISTA used in the proposed algorithm was run for the first ten iterations only. Moreover, to make the proposed algorithm more stable and computationally efficient, the bit-reversal order of subsets [28, 30] was applied instead of the traditional (sequential) order of subsets.

### 2.6. Simulation and Phantom Studies

**Figure 1:**
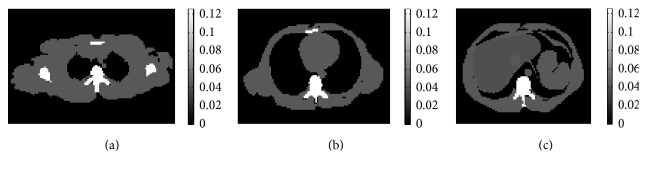
Three transverse slices from Zubal phantom displaying shoulder (a), chest (b), and abdomen (c).

In the simulation study, a cone-beam CT geometry with a source-to-isocenter distance of 100 cm and source-to-detector distance of 153.6 cm was modelled. The image object was a Zubal phantom [31] with 128 × 128 × 128 pixels and a voxel size of 0.208 cm.  Figure 1 illustrates three transaxial slices at the shoulder level, chest level, and abdomen level. A low-dose projection data (10k photons per detector bin) with Poisson noise was generated at 60 view angles equally spaced between 0° and 360°. The projection data had a dimension of 192 × 192 with a detector bin size of 0.213 × 0.213 cm^2^. The simulated phantom had attenuation coefficients of 0.604 cm^−1^, 0.193 cm^−1^, and 0.216 cm^−1^ for bone, liver, and soft tissue, respectively. To quantify the convergence speed of the reconstructed image, we used the relative root mean square error (RRMSE) defined as follows:

(12) $$\begin{array}{c} RRMSE=\sqrt{\frac{\sum_{j}{\left(\mu_{j}^{k,l+1}-\mu_{j}^{true}\right)}^{2}}{\sum_{j}{\left(\mu_{j}^{true}\right)}^{2}}}, \end{array}$$

where *μ*_*j*_^true^ is the true attenuation value at voxel *j*. We compared the proposed AOSTR-TDM-STF-FISTA algorithm to other algorithms including OSTR, AOSTR, OSTR-TDM-STF, OSTR-TDM-STF-FISTA, and AOSTR-TDM-STF.

In addition to simulation study, experimental data obtained from [32] were used to evaluate the performance of the proposed algorithm. A Catphan phantom (The Phantom Laboratory, Inc., Salem, NY) was scanned using the X-ray Volumetric Imager (XVI, Elekta Oncology Systems, Norcross, GA) with a typical setting of 120 kV, 40 mA, and 40 ms/frame. The distances of the X-ray source to the detector plane and to the center of rotation are 153.6 cm and 100 cm. The detector panel with a size of 40.96 × 40.96 cm^2^ consists of 1024 × 1024 elements each of which has a size of 0.4 × 0.4 mm^2^. Projection data were collected at 669 views uniformly distributed over 360 degrees. To simulate a low-dose CT acquisition, 60 projections evenly extracted from 669 projections were used to reconstruct CT image. The reconstructed image matrix is of 512 × 512 × 512 voxels with a voxel size of 0.52 mm.

## 3. Results

### 3.1. Simulation Study

**Figure 2:**
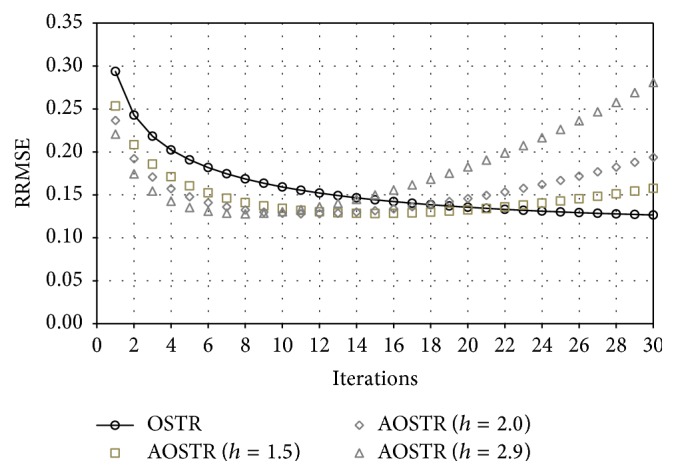
Simulation data: RRMSE values versus iteration numbers (30 subsets) for the OSTR and AOSTR (*h* = 1.5, 2.0, and 2.9) algorithms.

**Figure 3:**
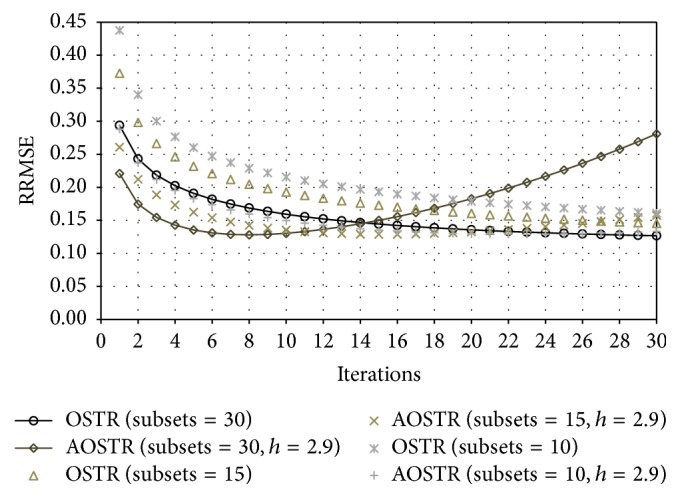
Simulation data: RRMSE values versus iteration numbers for OSTR and AOSTR with 10, 15, and 30 subsets. For AOSTR, *h* = 2.9 for all subsets.

To investigate whether OSTR can be accelerated by a power factor *h*, Figure 2 shows RRMSE values obtained using OSTR and AOSTR (*h* = 1.5, 2.0, and 2.9) at different iterations. We used 30 subsets for all algorithms. As seen in the comparison, AOSTR can reach lower RRMSE values faster than OSTR. This indicates that the present AOSTR algorithm can converge faster than the OSTR algorithm. The result also shows that the power *h* of the AOSTR algorithm can be up to 2.9. Furthermore, as illustrated in Figure 3, the present AOSTR algorithm (*h* = 2.9) provides faster reconstruction than the OSTR algorithm, regardless of numbers of subsets. However, due to the ill-posed nature of the reconstruction process, the image noise increases with the number of iterations. As a result, the RRMSE values quickly increase as the number of iterations is increased. In particular, the RRMSE value of the AOSTR algorithm with appropriate parameters increases earlier than others because it converges faster than the OSTR algorithm.

**Figure 4:**
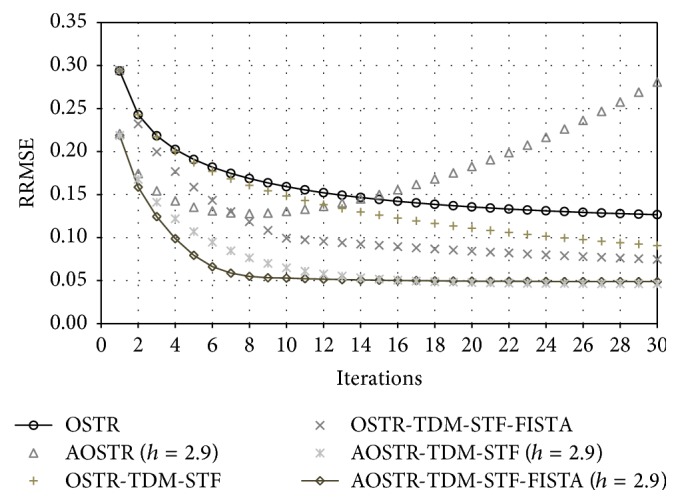
Simulation data: RRMSE values versus iteration numbers (30 subsets) for the OSTR, AOSTR (*h* = 2.9), OSTR-TDM-STF (*ω* = 0.0003), OSTR-TDM-STF-FISTA (*ω* = 0.0003), AOSTR-TDM-STF (*h* = 2.9 and *ω* = 0.001), and AOSTR-TDM-STF-FISTA (*h* = 2.9 and *ω* = 0.001) algorithms.

Figure 4 displays RRMSE values obtained from six algorithms: OSTR, AOSTR (*h* = 2.9), OSTR-TDM-STF (*ω* = 0.0003), OSTR-TDM-STF-FISTA (*ω* = 0.0003), AOSTR-TDM-STF (*h* = 2.9 and *ω* = 0.001), and AOSTR-TDM-STF-FISTA (*h* = 2.9 and *ω* = 0.001) at different iterations. We used 30 subsets for all algorithms. From the RRMSE plots, the AOSTR-TDM-STF-FISTA algorithm is obviously faster than all other algorithms. Also, both OSTR-TDM-STF-FISTA and AOSTR-TDM-STF outperform OSTR-TDM-STF. This indicates that the acceleration techniques can be used either before (i.e., AOSTR) or after (i.e., FISTA) the TDM-STF algorithm.

**Figure 5:**
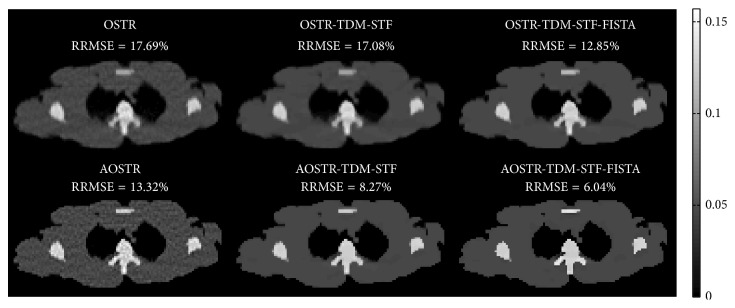
Simulation data: images reconstructed using the OSTR, OSTR-TDM-STF (*ω* = 0.0003), OSTR-TDM-STF-FISTA (*ω* = 0.0003), AOSTR (*h* = 2.9), AOSTR-TDM-STF (*h* = 2.9 and *ω* = 0.001), and AOSTR-TDM-STF-FISTA (*h* = 2.9 and *ω* = 0.001) algorithms. For each reconstructed image, we ran 8 iterations with 30 subsets.

**Figure 6:**
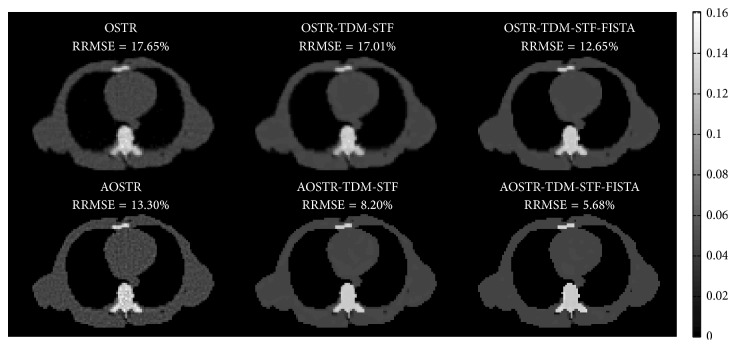
Same as Figure 5 but reconstructed from a different slice.

**Figure 7:**
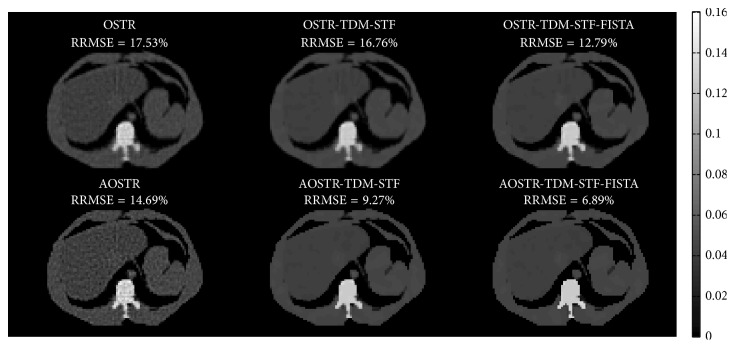
Same as Figure 5 but reconstructed from a different slice.

Figure 5 shows images of shoulder reconstructed using OSTR, AOSTR (*h* = 2.9), OSTR-TDM-STF (*ω* = 0.0003), OSTR-TDM-STF-FISTA (*ω* = 0.0003), AOSTR-TDM-STF (*h* = 2.9 and *ω* = 0.001), and AOSTR-TDM-STF-FISTA (*h* = 2.9 and *ω* = 0.001) algorithms. Each reconstruction was run with 8 iterations and 30 subsets. As compared with other reconstructions, the images from the proposed AOSTR-TDM-STF-FISTA algorithm are visually much closer to the true image, reflecting faster convergence. In addition, the AOSTR-TDM-STF algorithm is slightly slower than the AOSTR-TDM-STF-FISTA algorithm. Reconstruction images of chest and abdomen were also shown in Figures 6 and 7.

In Table 1, we compared the computational time required by each algorithm in C program on a Linux workstation with 64 GB memory and an eight-core Intel i7-5960 3.0 GHz CPU. Note that the computation time per iteration of AOSTR-TDM-STF-FISTA is slightly higher (i.e., 6%~10%) than that of OSTR, but it is minor as compared to the higher acceleration provided by the proposed method.

### 3.2. Phantom Study

**Figure 8:**
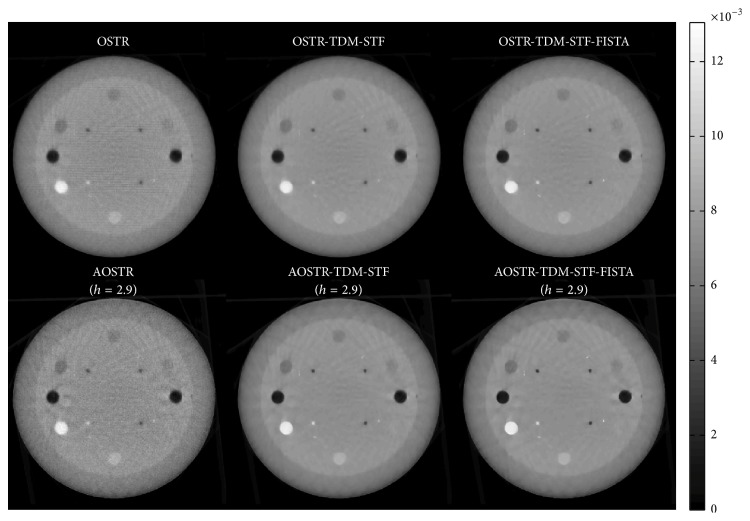
Catphan phantom data (contrast slice): images reconstructed using the OSTR, OSTR-TDM-STF (*ω* = 0.00003), OSTR-TDM-STF-FISTA (*ω* = 0.00003), AOSTR (*h* = 2.9), AOSTR-TDM-STF (*h* = 2.9 and *ω* = 0.0001), and AOSTR-TDM-STF-FISTA (*h* = 2.9 and *ω* = 0.0001) algorithms. For each reconstructed image, we ran 8 iterations with 30 subsets.

**Figure 9:**
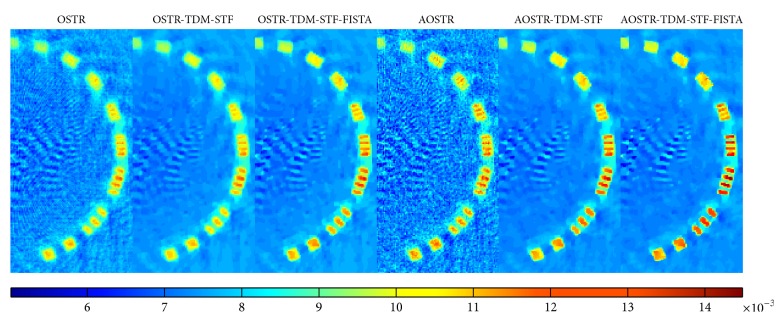
Same as Figure 4 but shows a zoomed-in resolution slice.

**Figure 10:**
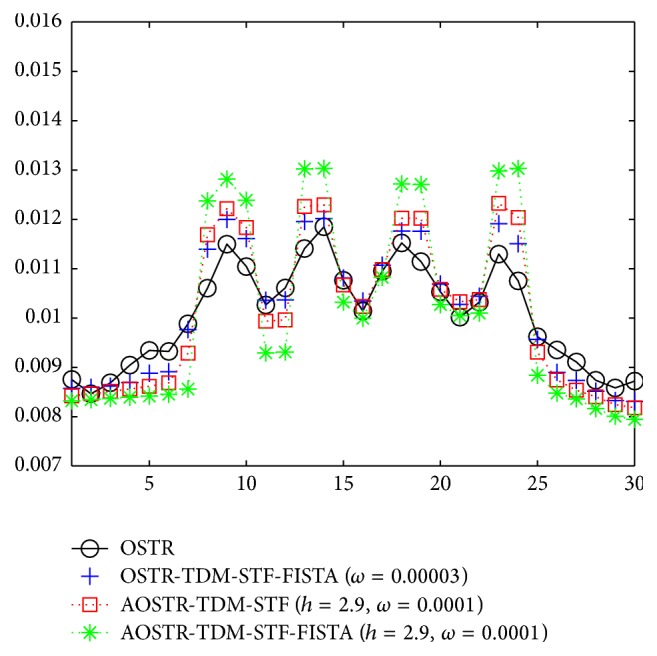
Profiles crossing the four line pairs for OSTR, OSTR-TDM-STF-FISTA (*ω* = 0.00003), AOSTR-TDM-STF (*h* = 2.9 and *ω* = 0.0001), and AOSTR-TDM-STF-FISTA (*h* = 2.9 and *ω* = 0.0001) reconstructions shown in Figure 9.

To investigate whether good image quality can be obtained after few iterations, we ran 8 iterations and 30 subsets for all algorithms. Figures 8 and 9 illustrate the contrast slice and the resolution slice (zoomed-in view), respectively, reconstructed using the OSTR, OSTR-TDM-STF (*ω* = 3 × 10^−5^), OSTR-TDM-STF-FISTA (*ω* = 3 × 10^−5^), AOSTR (*h* = 2.9), AOSTR-TDM-STF (*h* = 2.9 and *ω* = 0.0001), and AOSTR-TDM-STF-FISTA (*h* = 2.9 and *ω* = 0.0001) algorithms. It can be seen in Figure 8 that TDM-STF-based reconstruction algorithms can efficiently reduce noise. As shown in Figure 9, the images from accelerated algorithms are sharper compared with other images from unaccelerated algorithms (i.e., OSTR and OSTR-TDM-STF). Furthermore, Figure 10 shows that the present AOSTR-TDM-STF-FISTA algorithm can provide better resolution than the other two accelerated algorithms: OSTR-TDM-STF-FISTA and AOSTR-TDM-STF.

## 4. Summary and Discussion

Since the introduction of CT in 1970s, a wide variety of techniques have been developed to reduce radiation dose, scan time, and image reconstruction time while providing sufficient image quality for diagnosis. However, images reconstructed from low-dose CT data are degraded by noise and artifacts. To address this issue, many iterative reconstruction algorithms were proposed [13–15]. Over the past few years, TV has rapidly become a popular and powerful tool for low-dose CT image reconstruction [3–8]. In general, TV-based reconstruction problems are solved by a two-step alternating minimization scheme [3–8], including an iterative reconstruction algorithm for minimizing data discrepancy and a gradient-based search method for solving the TV regularization problem. Despite a substantial improvement in image quality, TV-based reconstruction methods suffer from high computational cost and slow convergence rate.

Motivated by this issue, we presented a CS-based reconstruction framework that combines several techniques to speed up the convergence rate of the two-step alternating minimization algorithm. First, we implemented the TDM-STF algorithm which was shown to be superior to conventional TV-based algorithms [6, 8]. Second, we combined TDM-STF with OSTR [13] instead of OS-SART [15]. Third, we applied the power acceleration scheme [16–18] and the FISTA algorithm [19] to OSTR and TDM-STF, respectively. To investigate whether combining the above-mentioned techniques can provide powerful acceleration for the two-step alternating minimization, we compared the performance of the proposed AOSTR-TDM-STF-FISTA algorithm and other algorithms including OSTR, AOSTR, OSTR-TDM-STF, OSTR-TDM-STF-FISTA, and AOSTR-TDM-STF.

In the simulation study, we showed the feasibility of combining the above-described acceleration techniques to improve the convergence rate of the two-step alternating minimization algorithm. The proposed AOSTR-TDM-STF-FISTA algorithm requires less iterations for convergence than all other algorithms. As also shown in Figures 5–7, the proposed AOSTR-TDM-STF-FISTA algorithm can provide better image quality compared with all other algorithms. We also evaluated the performance of the proposed method using experimental phantom data [32], which support the results observed in the simulation study. More importantly, the above-described acceleration techniques can greatly improve reconstruction speed with minor additional computational time (6%~10%). However, as the size of reconstructed images and projection data increases, the additional computational time can increase accordingly. Fortunately, image reconstruction using graphics processing unit can drastically reduce the reconstruction time [33–35].

Although the convergence proof for the proposed AOSTR-TDM-STF-FISTA algorithm is not available, it could rapidly achieve stable and lower RRMSE as compared with other presented algorithms. This suggests that the proposed AOSTR-TDM-STF-FISTA algorithm has the potential to satisfy the requirement of fast image reconstruction in practical CT applications. We also observed that the proposed AOSTR-TDM-STF-FISTA algorithm did not show any unstable convergence behavior. This may be due to the fact that the FISTA algorithm is terminated after the first ten iterations. In fact, we found out that it is not necessary to perform the FISTA algorithm per iteration. This is simply because the proposed AOSTR-TDM-STF-FISTA algorithm was observed to approach a stable solution after several iterations. Alternatively, the FISTA algorithm that combines with a diminishing step size [28] can be performed in each iteration while maintaining a stable convergence behavior. However, the adaptive step-size method requires additional parameters which may make image reconstruction more complicated.

In this study, we applied the power acceleration scheme to OS-type reconstruction algorithms. The highest acceleration can be achieved using the power factor of *h* = 2.9. This value is close to the upper limit (=3) found in previous results for emission tomography [16, 17] and might imply that the upper limit of the power *h* was not sensitive to imaging systems and other factors including the number of subsets, image objects, and noise levels [16–18]. In addition to the power factor *h*, the threshold value *ω* determines the reconstruction result and the convergence rate of the proposed algorithm. For simulated data, we adjusted the threshold value (*ω*) to generate the best performance in terms of RRMSE. For phantom data, the value of *ω* was adjusted in terms of lower image noise while maintaining acceptable spatial resolution similar to or better than that without TV. Unlike the previous studies [6, 8] that used the projected gradient method [36] to automatically determine *ω*, our study used fixed values of *ω*. This is because the automatic determination of the threshold would increase computation cost [8]. More importantly, dynamic adaptation of *ω* may not guarantee an optimal image quality [23]. An efficient optimization of the step size is necessary. This is beyond the goal of this study but will be studied in our future work.

In addition to the acceleration techniques used in this study, many techniques for improving the convergence rate of the two-step alternating minimization algorithm are available. For example, one can use improved TV-based reconstruction algorithms such as TV minimization with dual dictionaries [20], anisotropic TV minimization [21], WTD minimization [22], and TV minimization with half-threshold filtering [23]. However, combining these algorithms does not guarantee optimal results. For example, we had applied WTD [22] to our AOSTR-TDM-STF-FISTA algorithm, and there was a slight improvement in terms of image quality (data not shown). However, weighting values calculated using the neighboring voxels would increase computation time considerably, especially in three-dimensional cases. Despite the previous study [22] that showed the advantage of WTD over TD, combining our AOSTR-TDM-STF-FISTA algorithm with WTD may not be beneficial. More importantly, acceleration techniques that add extra parameters to the existing algorithm may lead to unstable and inaccurate results. Further study is possible on exploring this.

## 5. Conclusion

We have presented a CS-based reconstruction framework that combines several acceleration techniques. Both simulation and phantom studies show that, by using the proposed method, the convergence rate of the two-step alternating minimization algorithm can be improved substantially.

## Figures and Tables

**Pseudocode 1:**
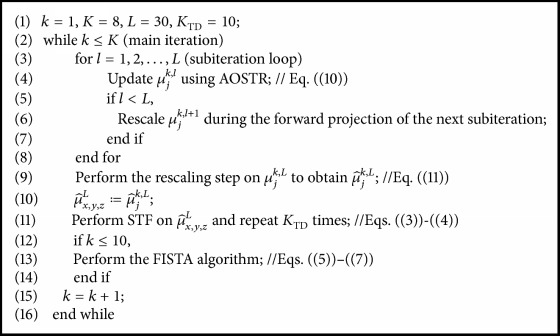


**Table 1:** Computational time for each algorithm.

Algorithms	Time (sec) per iteration	
	Subsets = 10	Subsets = 30
OSTR	9.87	10.19
AOSTR	10.56	10.52
OSTR-TDM-STF	10.06	10.38
OSTR-TDM-STF-FISTA	10.13	10.46
AOSTR-TDM-STF	10.76	10.71
AOSTR-TDM-STF-FISTA	10.88	10.80
